# Salinity and Heavy Metal Tolerance, and Phytoextraction Potential of *Ranunculus sceleratus* Plants from a Sandy Coastal Beach

**DOI:** 10.3390/life12121959

**Published:** 2022-11-23

**Authors:** Gederts Ievinsh, Zaiga Landorfa-Svalbe, Una Andersone-Ozola, Andis Karlsons, Anita Osvalde

**Affiliations:** 1 Department of Plant Physiology, Faculty of Biology, University of Latvia, 1 Jelgavas Str., LV-1004 Rīga, Latvia; 2 Institute of Biology, University of Latvia, 4 Ojāra Vācieša Str., LV-1004 Rīga, Latvia

**Keywords:** heavy metals, ion accumulation, nitrophily, phytoextraction potential, *Ranunculus sceleratus*, salinity tolerance

## Abstract

The aim of the present study was to evaluate tolerance to salinity and different heavy metals as well as the phytoextraction potential of *Ranunculus sceleratus* plants from a brackish coastal sandy beach habitat. Four separate experiments were performed with *R. sceleratus* plants in controlled conditions: (1) the effect of NaCl gradient on growth and ion accumulation, (2) the effect of different Na^+^ and K^+^ salts on growth and ion accumulation, (3) heavy metal tolerance and metal accumulation potential, (4) the effect of different forms of Pb salts (nitrate and acetate) on plant growth and Pb accumulation. A negative effect of NaCl on plant biomass was evident at 0.5 g L^−1^ Na^+^ and growth was inhibited by 44% at 10 g L^−1^ Na^+^, and this was associated with changes in biomass allocation. The maximum Na^+^ accumulation (90.8 g kg^−1^) was found in the stems of plants treated with 10 g kg^−1^ Na^+^. The type of anion determined the salinity tolerance of *R. sceleratus* plants, as Na^+^ and K^+^ salts with an identical anion component had a comparable effect on plant growth: nitrates strongly stimulated plant growth, and chloride treatment resulted in slight but significant growth reduction, but plants treated with nitrites and carbonates died within 4 and 5 weeks after the full treatment, respectively. The shoot growth of *R. sceleratus* plants was relatively insensitive to treatment with Mn, Cd and Zn in the form of sulphate salts, but Pb nitrate increased it. Hyperaccumulation threshold concentration values in the leaves of *R. sceleratus* were reached for Cd, Pb and Zn. *R. sceleratus* can be characterized as a shoot accumulator of heavy metals and a hyperaccumulator of Na^+^. A relatively short life cycle together with a high biomass accumulation rate makes *R. sceleratus* useful for dynamic constructed wetland systems aiming for the purification of concentrated wastewaters.

## 1. Introduction

Phytoremediation is an emerging sustainable technology for environmental restoration relying on the ability of vascular plants to promote the detoxification or removal of chemical contaminants. Among different specific approaches, phytoextraction of metal ions has gained particular scientific interest due to the enormous potential of the method for the practical restoration needs of contaminated degraded lands and waters [1,2,3].

Vascular plants need to possess a certain set of physiological characteristics in order to be considered for use in phytoextraction systems. Most importantly, in addition to the ability to accumulate metal ions in aerial parts at high concentration, plants need to be tolerant against the particular metal, but these two traits are independently encoded and controlled [4]. Therefore, the choice of the appropriate plant species with reasonable metal extraction potential is a critical stage in the establishment of a successful phytoextraction study. A commonly used approach includes a batch analysis of species native to a contaminated site, with chemical screening first in the field and then in controlled conditions [5,6]. In contrast to the high specificity of tolerance and accumulation of specialized ecotypes against particular metals usually found in these types of studies, it is reasonable to also search for plant species with general innate tolerance to heavy metals [7]. This type of tolerance is most likely to be found in highly heterogeneous habitats supporting the evolution of broadly adapted and phenotypically plastic plant species. 

A specific set of biogeochemical conditions in wetland ecosystems results in the rhizospheric accumulation of heavy metals [8,9]. It is logical to assume that wetland species have developed mechanisms to cope with high spatial and temporal heterogeneity in soil metal concentration. Therefore, species native to wetland [10] and saline habitats [11,12] as well as saline wetlands [9,13] have been considered to be useful resources in phytoremediation studies. Saline wetland species seem to be especially promising models for phytoextraction experiments, as many physiological mechanisms (efficient induction of active membrane transport systems, vacuolar sequestration of ions, maintenance of osmotic balance, enzymatic protection against endogenous oxidative stress, etc.) are shared among salt-tolerant and heavy-metal-tolerant plant species [11,14]. 

*Ranunculus sceleratus* L. is a semi-aquatic species of the Northern Hemisphere with circumpolar distribution often found in wetland habitats. The species is extremely resistant to soil flooding, with a survival strategy based on leaf petiole elongation to promote leaf blade contact with the aerial environment to sustain photosynthesis [15], and the constitutive presence of aerenchyma in the roots [16]. Although not specifically associated with a sea coast, *R. sceleratus* is commonly found in brackish coastal habitats: it is an umbrella species of the European protected habitats EUH 1310 “*Salicornia* and other annuals colonizing mud and sand” [17] and EUH 1640 “Boreal Baltic sandy beaches with perennial vegetation” [18]. According to the re-calibrated Ellenberg indicator values for British flora, *R. sceleratus* is characterized as a species occurring in both saline and non-saline habitats (indicator value 3 out of 9) on highly fertile soils with fertility status between rich and extremely rich (indicator value 8 out of 9) [19]. According to the recently designed ecological indicator values of Swedish vascular plants, *R. sceleratus* is favored by moderate salinity but not restricted to saline habitats (indicator value 3 out of 5) and is confined to extremely fertile soils, mostly artificially N-enriched ones (indicator value 9 out of 9) [20], suggesting the nitrophilic status of the species. 

No salinity tolerance and Na^+^ accumulation studies in controlled conditions have been performed with *R. sceleratus*, but in natural conditions of salt-affected coastal habitats, the species has been classified as moderately Na^+^ accumulating and a tight regulator of the electrolyte level in leaf tissues through inversely controlled changes in K^+^ and Na^+^ concentrations reflected by a wide range of tissue K^+^:Na^+^ concentration ratios [21]. 

*R. sceleratus* has been used in artificial wetland systems because of its ability to remove dissolved nitrogen and phosphorus [22]. In addition, the heavy metal accumulation potential of the species has been assessed in natural conditions, but the metal accumulation ability in shoots was relatively low in comparison to that in the roots [23]. Recently, *R. sceleratus* plants growing naturally on wastewater-affected soil [24] and in a river delta region [25] were analyzed for their phytoextraction potential. However, no studies have explored the heavy metal tolerance and accumulation potential of *R. sceleratus* in controlled conditions. 

The aim of the present study was to evaluate the tolerance to salinity and different heavy metals as well as phytoextraction potential of *R. sceleratus* plants from a brackish coastal sandy beach habitat. In particular, it was hypothesized that plants will have both a relatively high tolerance and metal accumulation potential.

## 2. Materials and Methods

### 2.1. Plant Material and Experiments

*Ranunculus sceleratus* L. plants growing naturally in a sea-water-affected wet sandy beach habitat on coast of the Riga Gulf of the Baltic Sea (Salacgrīva, Latvia; Figure S1) were used as a source of seeds used in the present study. Seeds were collected in August, kept at room temperature for one month and stored at 4 °C until used. 

Four separate experiments were performed with *R. sceleratus* plants in controlled conditions: (1) effect of NaCl gradient on growth and ion accumulation, (2) effect of different Na^+^ and K^+^ salts on growth and ion accumulation, (3) heavy metal tolerance and metal accumulation potential, (4) effect of different forms of Pb salts on plant growth and Pb accumulation (Table 1). 

### 2.2. Plant Establishment and Cultivation

Seeds were surface sterilized with water-diluted (50%) household bleach Ace (Procter & Gamble, Warszawa, Poland) for 7 min followed by washing in sterile deionized water (10 × 2 min). Seeds were sown in sterile plastic tissue culture containers with 2 cm autoclaved commercial garden soil (Biolan, Eura, Finland) mixed with sterile deionized water. Containers were placed in a plant growth cabinet MLR-352H (Sanyo Electric, Osaka, Japan), photoperiod 16 h (40 µmol m^−2^ s^−1^), day/night temperature 20/15 °C. After 25 days, seedlings were transplanted to 200 mL plastic containers filled with a mixture of quartz sand (Saulkalne S, Saulkalne, Latvia) and heat-treated (60 °C, 24 h) garden soil (Biolan, Eura, Finland) 1:3 (*v*/*v*). Containers were placed in 48 L plastic boxes closed with lids, placed in greenhouse and gradually adapted to greenhouse conditions. Experimental automated greenhouse (HortiMaX, Maasdijk, The Netherlands) was used for the study during winter–spring season. Supplemented light was provided by Master SON-TPIA Green Power CG T 400 W (Philips, Amsterdam, The Netherlands) and Powerstar HQI-BT 400 W/D PRO (Osram, Munich, Germany) lamps (photon flux density of photosynthetically active radiation 380 µmol m^−2^ s^−1^ at the plant level), 16 h photoperiod. Day/night temperature was 22/15 °C, relative air humidity was maintained at 60 to 70%. After 28 days, seedlings were transplanted to 0.5 L plastic containers filled with mixture of quartz sand (Saulkalne S, Saulkalne, Latvia) and garden soil (Biolan, Eura, Finland) 1:1 (*v*/*v*). Soil was moistened with deionized water. Substrate water content was monitored with HH2 moisture meter equipped with WET-2 sensor (Delta-T Devices, UK) and maintained not less than 60% throughout the experiment using deionized water. Individual containers were randomly placed on greenhouse bench and were repositioned once a week. Every week plants were fertilized with Yara Tera Kristalon Red and Yara Tera Calcinit fertilizers (Yara International, Oslo, Norway). A stock solution was prepared for each fertilizer (100 g L^−1^) and working solution contained 25 mL of each per 10 L deionized water, used with a rate 50 mL per container.

### 2.3. Treatments

Five individual plants were randomly selected for each treatment in each of four separate experiments. For salinity treatment in Experiment 1, plants were gradually treated with NaCl solution in deionized water during 3 weeks, until final concentration of Na^+^ in substrate was reached (Table 1). Plants were cultivated for additional 5 weeks after the treatment was completed. For salinity treatment in Experiment 2, plants were gradually treated with respective salt solution during 2 weeks until final concentration of Na^+^ (4.0 g L^−1^ substrate) and K^+^ (6.8 g L^−1^ substrate) was reached. Plants were cultivated for additional 5 weeks after the treatment. For heavy metal treatment in Experiment 3, plants were gradually treated with respective salt solutions during 2 weeks until the planned final concentration in substrate was reached for the particular treatment (Table 1). Plants were cultivated for additional 5 weeks after the full treatment. Similarly, in Experiment 4, plants gradually received final concentration of Pb in substrate by using either Pb(NO_3_)_2_ or Pb(CH_3_COO)_2_ solution. Plants were cultivated for additional 7 weeks after the full treatment was reached.

### 2.4. Termination of Experiments and Measurements

At the termination of experiments, plant shoots were cut and rinsed with deionized water. Individual plants were separated in rosette leaves (their petioles and blades), stems, stem leaves, inflorescences (flowers) as well as small leaves at the base of rosette if these were distinguishable. In Experiment 2, senescent (dry) leaves and live leaves were handled separately. Roots were thoroughly washed with tap water to remove any adhered soil particles and rinsed with deionized water. Both fresh and dry mass (after drying at 60 °C for 72 h) of individual plant parts were measured. 

In plant material from Experiments 1 and 2, Na^+^, K^+^ and electrical conductivity (EC) measurements were performed in five individual samples per treatment from individual biological replicates. Plant tissues were homogenized by crushing by hand and a sample (0.2 g) was taken. Tissues were ground with mortar and pestle to a fine powder and 10 mL of deionized water was added. The homogenate was stirred with pestle for 1 min. After filtration through nylon mesh cloth (No. 80), homogenate was used for measurement of ion concentration by LAQUAtwin compact meters B-722 (Na^+^) and B-731 (K^+^), and electrical conductivity by LAQUAtwin conductivity meter B-771 (Horiba, Kyoto, Japan). Three analytical replicates were performed for each sample and the average value was calculated, expressed on dry biomass basis.

Concentrations of Cd, Mn, Pb and Zn were measured in dried material for all plant parts. For each plant sample, approximately 1 g of dried plant tissues were collected. Samples were ground and plant tissue test solution was prepared by dry ashing with HNO_3_ vapor and re-dissolving in a 3% HCl solution. The testing solution was used for the determination of analyzed heavy metals. Microwave plasma atomic emission spectrometry (4200 MP-AES, Agilent, Santa Clara, CA, USA) was used for the measurement of metals according to manufacturer’s instructions. Analyzed element concentrations in plant tissue were expressed as mg kg^−1^ dry mass. 

Bioconcentration factor was calculated as a ratio between tissue concentration of the respective metal and the initial concentration of the metal in soil.

### 2.5. Data Analysis

Results were analyzed by KaleidaGraph (v. 5.0, Synergy Software, Reading, PA, USA). Statistical significance of differences for measured parameters between treatments and accessions was evaluated by one-way ANOVA using post hoc analysis with minimum significant difference. Significant differences were indicated at *p* < 0.05.

## 3. Results

### 3.1. Experiment 1: NaCl Gradient

**Figure 1:**
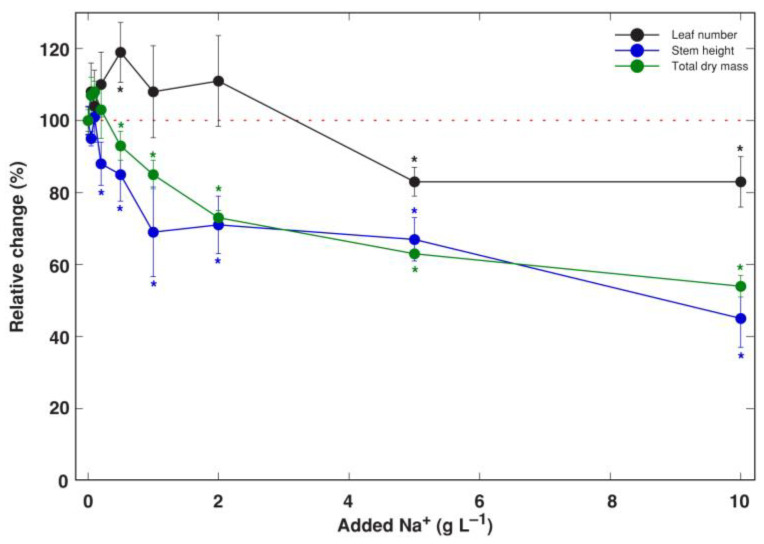
Relative effect of Na^+^ (in a form of NaCl) in substrate on growth of *Ranunculus sceleratus* plants. Plants were cultivated for 5 weeks after the full treatment. Data are means from 5 replicates ± SE. Asterisks indicate statistically significant difference from control (*p* < 0.05).

**Figure 2:**
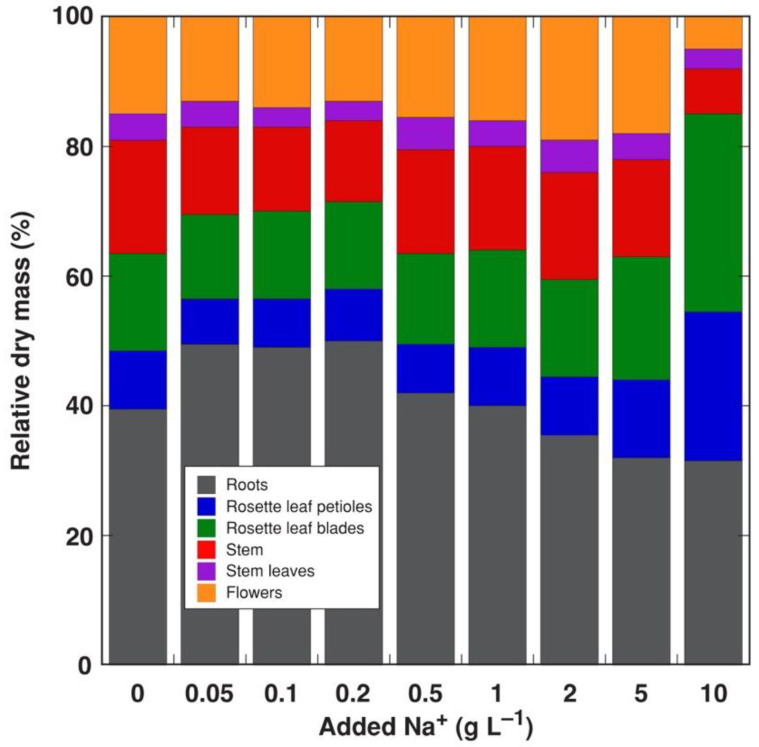
Effect of Na^+^ (in a form of NaCl) on relative dry mass distribution in *Ranunculus sceleratus* plants. Plants were cultivated for 5 weeks after the full treatment.

For *R. sceleratus* plants cultivated in the presence of increasing substrate NaCl concentration (Figure S2), there was a slight but significant increase in the number of leaves at 0.5 g L^−1^ Na^+^, but the total dry mass did not show a significant increase (Figure 1). A negative effect of increasing NaCl on the stem height was evident already at 0.2 g L^−1^ Na^+^, and on total biomass at 0.5 g L^−1^ Na^+^. The number of leaves significantly decreased for plants treated with 5 and 10 g L^−1^ Na^+^. Biomass accumulation was inhibited by 44% at 10 g L^−1^ Na^+^, and this was associated with changes in biomass allocation, as these plants had proportionally lower allocation to generative structures and roots, and higher to leaves (Figure 2). 

**Figure 3:**
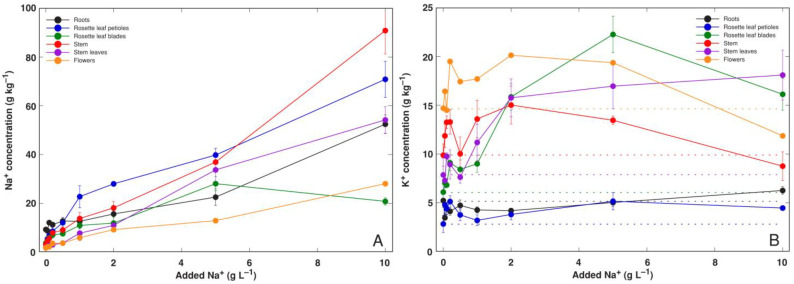
Accumulation of Na^+^ (**A**) and K^+^ (**B**) in different parts of *Ranunculus sceleratus* plants dependent on concentration of Na^+^ (in a form of NaCl) in a substrate. Plants were cultivated for 5 weeks after the full treatment. Data are means from 5 replicates ± SE. Dotted lines in B indicate control values.

In control plants, the highest Na^+^ concentration was found in the roots, reaching 9.3 g kg^−1^ (Figure 3A). In the blades of rosette leaves it was 3.7 g kg^−1^, followed by the petioles of rosette leaves (3.3 g kg^−1^), stems (3.1 g kg^−1^), stem leaves (1.8 g kg^−1^) and flowers (1.7 g kg^−1^). Tissue Na^+^ concentration increased in all plant parts with increasing substrate Na^+^ level, but the character of the response was different for various parts (Figure 3). The most pronounced increase in Na^+^ concentration at a low to moderate external NaCl level was seen for leaf petioles, followed by that for the stem, stem leaves and roots. Na^+^ concentration in the leaf blades increased with increasing substrate Na^+^ similar to that in the roots up to 5 g L^−1^, but decreased in plants treated with 10 g L^−1^ Na^+^ to a level even lower than that in flowers, where there was the lowest Na^+^ concentration in all other treatments. The maximum Na^+^ concentration was found in the stems of plants treated with 10 g kg^−1^ Na^+^, reaching 90.8 g kg^−1^. NaCl deposition on the leaf surface in a crystalline form was evident for *R. sceleratus* plants at the highest substrate NaCl (Figure S3).

The bioconcentration factor for Na^+^ was the highest in the roots of control plants, and it decreased in an order roots > rosette leaf petioles > rosette leaf blades > stem > stem leaves > flowers (Table 2). In all plant parts, the bioconcentration factor decreased with increasing substrate Na^+^ concentration. However, the decrease was not equally rapid in all parts, and at moderate salinity (1–2 g Na^+^ L^−1^), the most efficient accumulation occurred in rosette leaf petioles. The most efficient accumulation at high salinity (5–10 g Na^+^ L^−1^) was evident for rosette leaf petioles, the stem and stem leaves.

**Figure 4:**
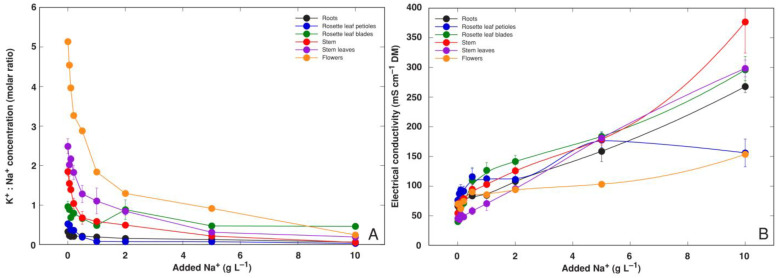
Changes in K^+^: Na^+^ molar concentration ratio (**A**) and electrical conductivity (**B**) in different parts of *Ranunculus sceleratus* plants dependent on concentration of Na^+^ (in a form of NaCl) in a substrate. Plants were cultivated for 5 weeks after the full treatment. Data are means from 5 replicates ± SE.

K^+^ concentration significantly increased in the rosette leaf blades, stems, stem leaves and flowers in all Na^+^ treatments up to 5 g L^−1^ but remained relatively low in the roots and rosette leaf petioles (Figure 3B). The K^+^:Na^+^ molar concentration ratio decreased with increasing substrate NaCl level, but the trend was clearly organ-specific (Figure 4A). Even for control plants, the roots and rosette leaf petioles had K^+^:Na^+^ values below 0.5, and it was about 1 in rosette leaf blades. A higher ratio was characteristic for the stem and stem leaves, and for flowers. Tissue EC increased gradually and relatively similarly in all plant parts with increasing substrate Na^+^ concentration except in flowers and rosette leaf blades at the highest treatment concentration (Figure 4B). Similar to Na^+^ concentration, EC was the highest in the stems of plants treated with 10 g L^−1^ Na^+^ (378 mS cm^−1^). 

### 3.2. Experiment 2: Na^+^ and K^+^ Salts

**Figure 5:**
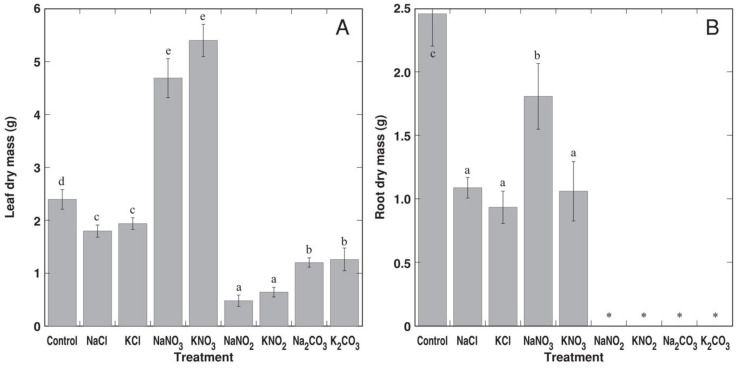
Effect of different Na and K salts on growth of shoots (**A**) and roots (**B**) of *Ranunculus sceleratus* plants. *, roots were decayed. Plants were cultivated for 5 weeks after the full treatment. Data are means from 5 replicates ± SE. Different letters indicate statistically significant differences between treatments (*p* < 0.05).

In the conditions of Experiment 2, *R. sceleratus* plants did not form flowers. Plants treated with NaNO_2_ and KNO_2_ started to show signs of decline 2 weeks after full treatment and became dry 4 weeks after full treatment (Figure S4). Plants treated with Na_2_CO_3_ and K_2_CO_3_ started to show signs of decline 4 weeks after full treatment and became dry 5 weeks after full treatment (Figure S4). The roots of these plants fully decayed before termination of the experiment, and they had the lowest leaf biomass (Figure 5). While leaf growth was significantly inhibited by treatment with NaCl and KCl (by 25 and 19%, respectively), leaf biomass increased by 96 and 125% in plants treated with NaNO_3_ and KNO_3_, respectively. However, the biomass of the roots was significantly inhibited by NaCl, KCl, NaNO_3_ and KNO_3_ treatment.

**Figure 6:**
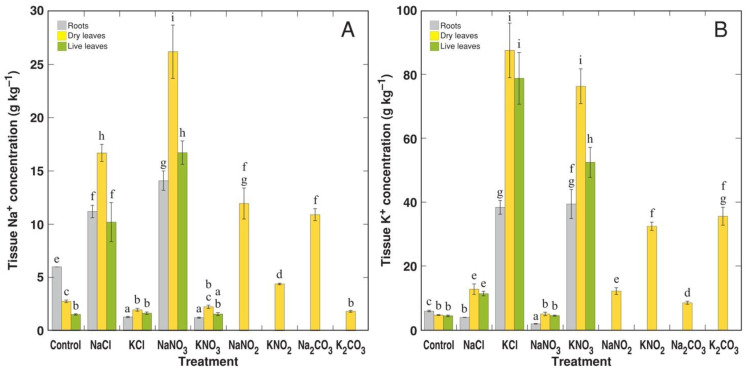
Effect of different Na and K salts on accumulation of Na^+^ (**A**) and K^+^ (**B**) in different parts of *Ranunculus sceleratus* plants. Plants were cultivated for 5 weeks after the full treatment. Data are means from 5 replicates ± SE. Different letters indicate statistically significant differences between treatments (*p* < 0.05).

**Figure 7:**
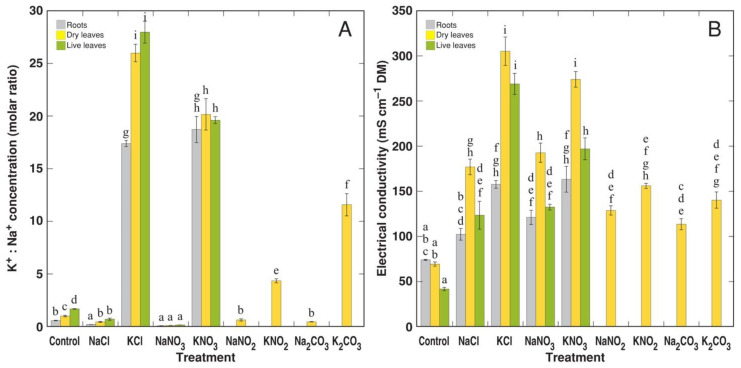
Effect of different Na and K salts on accumulation of K^+^:Na^+^ molar concentration ratio (**A**) and electrical conductivity (**B**) in different parts of *Ranunculus sceleratus* plants. Plants were cultivated for 5 weeks after the full treatment. Data are means from 5 replicates ± SE. Different letters indicate statistically significant differences between treatments (*p* < 0.05).

The highest concentration of Na^+^ accumulated in the dry leaves of plants treated with NaNO_3_, followed by those of NaCl-treated plants (Figure 6A). Less Na^+^ accumulated in the dry leaves of plants treated with NaNO_2_ and Na_2_CO_3_. However, the dry leaves of KCl- and KNO_3_-treated plants accumulated equal concentrations of K^+^, with significantly lower levels in plants treated with KNO_2_ and K_2_CO_3_ (Figure 6B). As a result of the dominant accumulation of Na^+^ or K^+^ in plants treated with Na^+^ or K^+^ salts, the K^+^:Na^+^ molar concentration ratio showed extremely high variability in spite of the identical morphological appearance of plants receiving the same anion component (Figure 7A). Interestingly, tissue EC was significantly higher in plants treated with K^+^ salts in comparison to plants treated with Na^+^ salts (Figure 7B). Differences in EC between NaNO_2_ and KNO_2_, and Na_2_CO_3_ and K_2_CO_3_ treatments were less pronounced. The highest electrolyte concentration was seen in dry leaves, with similar concentrations in the roots and live leaves.

### 3.3. Experiment 3: Heavy Metals

**Figure 8:**
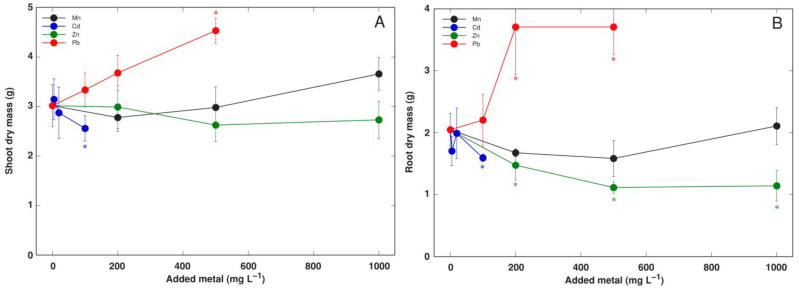
Effect of increasing concentration of heavy metals in substrate (in a form of MnSO_4_, CdSO_4_, ZnSO_4_ and Pb(NO_3_)_2_) on shoot (**A**) and root (**B**) dry mass of *Ranunculus sceleratus* plants. Plants were cultivated for 5 weeks after the full treatment. Data are means from 5 replicates ± SE. Asterisks indicate statistically significant (*p* < 0.05) differences from control.

Shoot growth of *R. sceleratus* plants was relatively insensitive to heavy metal treatment (Figure S5 and Figure 8A). Significant biomass reduction was evident only for plants treated with 100 mg L^−1^ Cd, but treatment with 500 mg L^−1^ Pb (in a form of Pb(NO_3_)_2_) significantly stimulated shoot growth. Root growth was sensitive to 100 mg L^−1^ Cd and all Zn treatments, but it was significantly stimulated by 200 and 500 mg L^−1^ Pb treatment (Figure 8B). 

**Figure 9:**
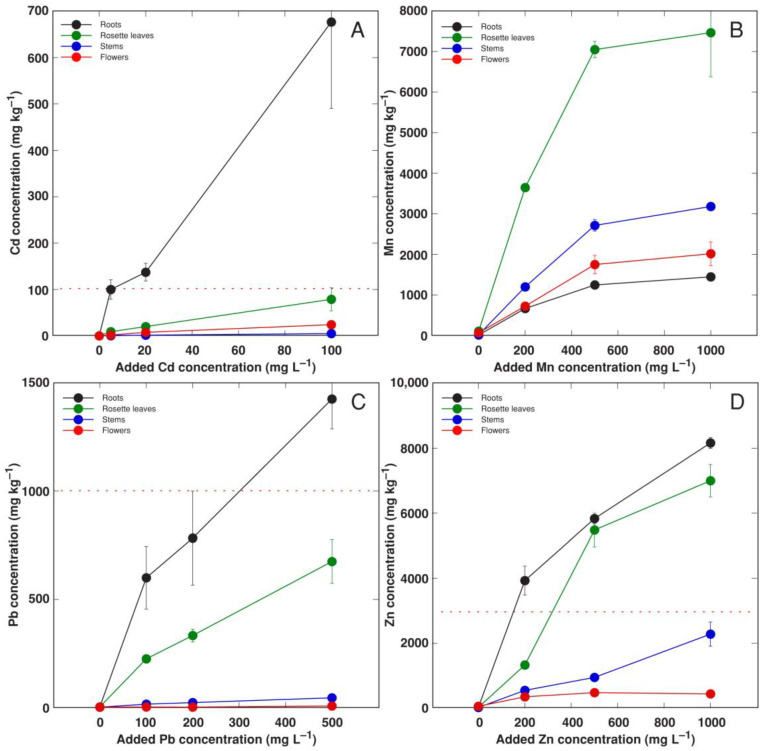
Effect of increasing concentration of Cd (**A**), Mn (**B**), Pb (**C**) and Zn (**D**) in substrate on accumulation of heavy metals in different parts of *Ranunculus sceleratus* plants. Plants were cultivated for 5 weeks after the full treatment. Data are means from 3 samples ± SE. Dashed line indicates threshold level of hyperaccumulation for the respective heavy metal.

Cd was preferentially accumulated in plant roots at an extremely high concentration (700 mg kg^−1^), but the hyperaccumulation threshold was also reached for leaves at the highest substrate Cd concentration (Figure 9A). Cd accumulation potential in the stems and flowers was very low. Mn preferentially accumulated in the rosette leaves of *R. sceleratus* plants, with a significantly lower concentration in stems, followed by generative structures, and with the lowest concentration in roots (Figure 9B). The maximum concentration of Mn in leaves (7 g kg^−1^) was reached already at 500 mg L^−1^ Mn. The highest concentration of Pb was also observed in plant roots, with the level in leaves being about half of that (Figure 9C). The concentration of Pb in generative parts was only negligible. In contrast, the accumulation potential of Zn was relatively similar in the roots and leaves, with somewhat higher levels in roots (Figure 9D). The hyperaccumulation threshold concentration for Zn accumulation was exceeded at 200 mg L^−1^ for roots and 500 mg L^−1^ for leaves. A significantly lower level of Zn accumulated in the stems, and this metal was almost completely excluded from generative parts. 

For all metals, their bioconcentration factors decreased with the increase in substrate metal concentration (Table 3). The highest bioconcentration factor in roots was for Cd and Zn, and in leaves for Mn and Zn. In the stem, only Mn and Zn had bioconcentration factors > 1.

### 3.4. Experiment 4: Forms of Pb Salts

**Figure 10:**
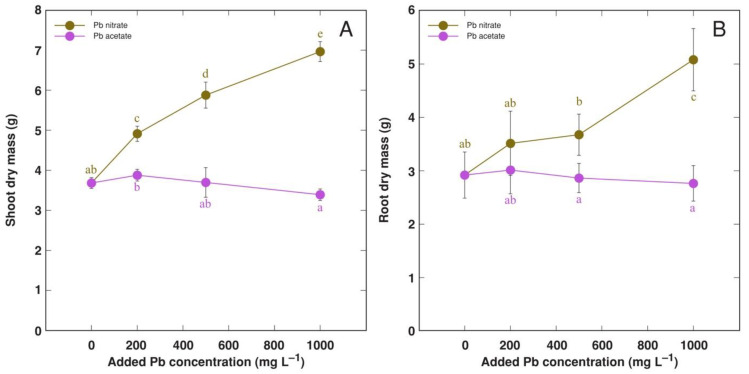
Effect of increasing concentration of Pb in substrate in a form of acetate or nitrate on shoot (**A**) and root (**B**) dry mass of *Ranunculus sceleratus* plants. Plants were cultivated for 7 weeks after the full treatment. Data are means from 5 replicates ± SE. Different letters indicate statistically significant differences between treatments (*p* < 0.05).

**Figure 11:**
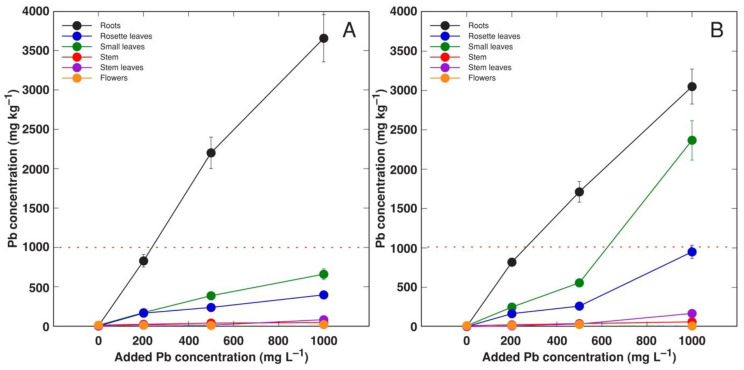
Effect of increasing concentration of Pb in substrate in a form of nitrate (**A**) or acetate (**B**) on accumulation of Pb in different parts of *Ranunculus sceleratus* plants. Plants were cultivated for 7 weeks after the full treatment. Data are means from 3 samples ± SE. Dashed line indicates threshold level of hyperaccumulation for Pb.

When the effect of Pb in a form of nitrate or acetate on the growth of *R. sceleratus* plants was compared, it was evident that nitrate-treated plants showed more vigorous growth and greener leaves in comparison to that in acetate-treated plants (Figure S6). The total dry mass of shoots under the Pb nitrate treatment, but not in the Pb acetate treatment, increased significantly in a concentration-dependent manner, confirming the nitrophilous nature of the species (Figure 10A). In addition, root growth was significantly stimulated at 1000 mg L^−1^ Pb in a form of nitrate (Figure 10B). Small rosette leaves in Pb acetate-treated *R. sceleratus* plants accumulated as much as 3 g kg^−1^ Pb, with the respective level in large rosette leaves reaching the hyperaccumulation threshold at 1000 mg kg^−1^ Pb in substrate (Figure 11B). Pb accumulation potential in the shoots of nitrate-treated plants was comparatively less pronounced (Figure 11A).

Bioconcentration factors for Pb were even smaller than those found in the previous experiment (Table 4). In roots, treatment with both Pb salts resulted in relatively similar bioconcentration factors. However, in leaves, Pb in the form of acetate showed more efficient bioconcentration ability in comparison to the nitrate form. 

## 4. Discussion

There are two physiological characteristics of plants that make them important in the context of heavy metal phytoremediation: metal tolerance and metal accumulation potential. In contrast to common assumptions, these traits are genetically independent and each is controlled by multiple genes [4]. Mechanisms of metal (hyper)tolerance and (hyper)accumulation in plants have been described in detail in a number of recent exhaustive reviews [26,27,28,29,30,31,32,33], and, therefore, will not be analyzed here. 

While many metal-tolerant species are metal excluders, the majority of metal accumulators have high tolerance to the specific metal(s) they are accumulating. Metal-accumulating plants are not as rare in nature as expected according to the ecological criteria of hyperaccumulation, which involves looking for them only in soils with increased metal content. The astonishing speed of local genetic adaptation, in some cases as little as 5 years [34], suggests that the genetic basis for metal tolerance and accumulation is possibly conserved in many species. Thus, genetically diverse *Armeria maritima* ecotypes native to metal-rich soils are known to have high metal tolerance and accumulation potential (aka *A. maritima* subsp. *halleri* s.l.) [35]. However, other subspecies of *A. maritima* from non-metalliferous habitats are also known to possess both heavy metal tolerance and high accumulation potential for a variety of metals [36]. On the other hand, species-wide heavy metal tolerance so far has been found only for a limited number of species [37,38], and it seems that many species are still to be found in the near future. For example, recent studies have helped to replenish the ranks of metal-resistant and highly accumulating species with *Bidens pilosa* (accumulating Cd) [39], *Celosia argentea* (accumulating Cd and Mn) [40], *Gossia fragrantissima* and *Gossia punctata* (accumulating Mn, Co, Zn) [41], and *Impatiens glandulifera* (accumulating Cd) [42].

The establishment of phylogenetic association between metal hyperaccumulation and salt tolerance in plants [43] further encourages and justifies further research in this direction [12]. Recently, we tried to look at the problem of Na^+^ accumulation in halophytes from the point of view of metal hyperaccumulation, defining possible threshold concentrations for defining electrolytophytic species and Na^+^ hyperaccumulators, based on analysis in field conditions [21]. In the present study, we assessed tolerance to and accumulation of both Na^+^ and heavy metals in a potentially nitrophilic coastal plant species, *R. sceleratus*, in controlled conditions. 

The tolerance of *R. sceleratus* plants to analyzed heavy metals Cd, Mn, Pb and Zn was very high. There were no visible symptoms of toxicity, and shoot growth was significantly inhibited only by the 100 mg L^−1^ Cd treatment (Figure 8A). However, root growth was inhibited by both Cd (200 mg L^−1^) and Zn (at all concentrations) (Figure 8B). *R. sceleratus* plants were especially tolerant to Pb as Pb(NO_3_)_2_ treatment even resulted in significant growth stimulation at both shoot and root level (Figure 8 and Figure 10). 

To characterize an ability to accumulate heavy metals, one of the criteria for defining the plant as a hyperaccumulator is the so-called hyperaccumulation threshold, a concentration of a particular metal in the shoots of the plant of interest [44]. In fact, the hyperaccumulation threshold concentration value for a particular metal does not have much relevance for studies in controlled conditions involving the cultivation of plants in artificially contaminated substrates. However, these values can be successfully used as performance indicators of the high metal accumulation ability of the particular plant species. How could the metal accumulation capacity of *R. sceleratus* be assessed in this context? Most importantly, the particular threshold values in the leaves of *R. sceleratus* were reached for Cd, Pb and Zn, and were relatively close to the value for Mn (Figure 9 and Figure 11). 

Especially striking was the ability of *R. sceleratus* to accumulate Pb in its leaves. Plant shoot uptake of Pb is often restricted by its low soil availability as well as anatomical barriers in plant roots [29,32]. As no means of artificial solubilization of Pb were used in the present study, it seems that the high accumulation of Pb in leaves could be due to the lack of physiological mechanisms restricting its transport to aboveground parts. Moreover, metal solubilization in the soil by root exudates cannot be ruled out, as shown for other halophytic species [13].

Another parameter for the comparison of metal accumulation ability is the bioconcentration factor (sometimes indicated as “bioaccumulation factor”), which shows the ratio between the soil metal concentration and the metal concentration taken up by the plant and accumulated in a certain part [26]. It is well-known that the bioconcentration factor for a particular metal is highly genotype-dependent, and it usually decreases with increasing substrate metal concentration [45]. The bioconcentration factors found in the present study for the roots of *R. sceleratus* (Table 3) were relatively similar to the ones established in field conditions [23]. However, in that study, all analyzed metals preferentially accumulated in plant roots, but differential accumulation was evident in the present study.

In addition to metal accumulation capacity, the growth rate and biomass of individual plants are important characteristics for consideration of phytoextraction potential. As traditional hyperaccumulator species usually have biomass 1–2 orders of magnitude lower than that of non-hyperaccumulating crop species [45], the overall phytoextraction potential of hyperaccumulators can be rather low. As nitrophilous species, *R. sceleratus* can form a relatively large biomass at high N availability, which might be an advantage when using it in wastewater purification systems with a high N load. 

The results from Experiment 2 clearly showed that the type of anion was important in determining the salinity tolerance of *R. sceleratus* plants, as Na^+^ and K^+^ salts with identical anion components had a comparable effect on plant growth (Figure 5). A similar effect of Na^+^ and K^+^ salts on a number of relatively salt-tolerant coastal plant species has been established recently [46,47]. In respect to anion type, nitrates strongly stimulated plant growth, and chloride treatment resulted in slight but significant growth reduction, but plants treated with nitrites and carbonates died within 4 and 5 weeks after the full treatment, respectively (Figures S4 and S5).

The alkalinity of carbonates and bicarbonates are usually considered the main reason for their more negative effect on plants in comparison to chlorides, which are neutral compounds [48,49]. However, recent evidence shows that high soil pH alone cannot explain the higher toxicity of alkaline salts, NaHCO_3_ and Na_2_CO_3_, in comparison to that of NaCl [50]. One of the reasons for the higher toxicity of saline–alkali soils for glycophyte species is considered to be the formation of proton-deficiency-inhibiting Na^+^/H^+^ antiport transport systems further leading to Na^+^ accumulation in plant tissues [51,52]. However, this should not be a problem for Na^+^-accumulating halophyte species; therefore, additional mechanisms are likely to exist for the higher toxicity of alkaline salts in these species. Indeed, the high sensitivity of *R. sceleratus* plants to nitrite and carbonate was not associated with the increased accumulation of Na^+^ or decrease in K^+^ (Figure 6). 

To understand the results of the present study, instead of the simple chemical nature of cation or anion, their physiological relevance in plants needs to be considered. In aqueous media in the presence of protons, carbonate converts to bicarbonate, but bicarbonate can dissociate at low proton concentration [53]. Therefore, differences in their effects might be indistinguishable and highly dependent on pH. Bicarbonate in plants is readily formed by carbonic anhydrases, in further participating in the carboxylation of acetyl-CoA to malonyl-CoA or the carboxylation of phosphoenolpyruvate to oxalacetate [54]. No studies have aimed to understand aspects of the carbonate/bicarbonate toxicity in plants apart from the different pH effects. However, a study with saline lake bacteria, performed with different Na^+^ salts at identical pH, showed that anion type predicts salinity responses and that sodium carbonate represents the strongest selective force [55].

Nitrate is a major plant source of nitrogen in soil [56]. However, plant species differ in their relative need for nitrogenous compounds. Based mostly on vegetation studies, a nitrophily has been defined as a need of the species for a relatively higher soil nitrate concentration in comparison to other plants [57]. However, sometimes the term is used merely to indicate increased tolerance to high nitrate availability in comparison to other species. In practice, the term has been used in ecology and vegetation science to indicate plant taxa natively associated exclusively only with very nitrogen-rich or artificially nitrogen-enriched soils. Plant nitrophily has not been much assessed from a physiological point of view. Only isolated attempts have been made to experimentally quantify nitrophily [58]. The effects of surplus nitrate have rarely been studied apart from the accumulation of nitrate in plant tissues, which occurs in situations unfavorable for the photosynthesis-dependent assimilation of nitrate to ammonium [59]. 

In plants, nitrite is an intermediate product of light-dependent nitrate reduction to ammonium [60]. However, exogenous nitrite is considered to be toxic for plants, and only relatively slowly has evidence been accumulated that nitrite itself can be used as a source of nitrogen in low nitrate conditions [61]. In contrast to animals, where nitrite toxicity is associated with the formation of methemoglobin [62], the mechanisms of nitrite toxicity in plants are far from clear. Growth reduction, the destruction of cellular structures and the inhibition of nutrient uptake are among the major negative effects of elevated nitrite exposure [63]. The conversion of nitrite to HNO_2_ in the soil root zone, accelerated by decreasing pH, has been associated with nitrite toxicity [64]. A tolerable concentration of nitrite in the soil is below 200 mg kg^−1^, or even below 75 mg kg^−1^ for sensitive plant species, as 225 mg NO_2_-N already resulted in a 55% biomass reduction in *Zea mays* [65]. However, in the present study, the nitrite concentration used was relatively much higher.

The present results experimentally confirmed the nitrophilic status of *R. sceleratus*, postulated based on the characteristic appearance of individuals in nitrogen-rich habitats [20]: nitrate as both sodium and lead salt resulted in the significant stimulation of biomass accumulation. When using Pb in a form of nitrate, treatment with increasing Pb doses is inevitably associated with increasing nitrate concentration in substrate. Therefore, any growth stimulation or other morphological changes due to Pb nitrate treatment could indicate a positive response to increasing nitrate availability. In such cases, it is correct to use different Pb salts or additional control compensating increased nitrogen availability [66].

## 5. Conclusions

According to a recently established concept [27], *R. sceleratus* can be characterized as a “shoot accumulator” of heavy metals, instead of being designated as a “hyperaccumulator”. Most importantly, the feature of high accumulation was achieved during cultivation in substrate without any means of increase in metal availability. However, the particular accession of *R. sceleratus* is also a Na^+^ hyperaccumulator, according to the criteria derived from a study with a large number of coastal plant species [21]. The plant appears to be hypertolerant to Na^+^ and K^+^ chloride and nitrate salinity, and relatively well-tolerant to nitrite and carbonate salinity.

From a theoretical point, *R. sceleratus* seems to be an extremely promising model species for studies of salinity and metal tolerance mechanisms, as well as electrolyte and metal accumulation potential. From a practical point, both tolerance and accumulation potential to different soil inorganic contaminants form the basis for various practical developments. The nitrophilic and amphibic character of the species indicates a potential for the remediation of soils and waters contaminated with nitrogen compounds. The relatively short life cycle together with a high biomass accumulation rate on the background of high nitrogen availability makes *R. sceleratus* useful for dynamic constructed wetland systems aimed at the purification of concentrated wastewaters. 

## Figures and Tables

**Table 1:** Experiments performed with *Ranunculus sceleratus* plants.

Experiment No.	Description	Treatments (Final Concentrations in Substrate)	Duration of Experiment
1	Effect of NaCl gradient	NaCl (equivalent to 0.05, 0.1, 0.2, 0.5, 1, 2, 5, 10 g L^−1^ Na^+^)	Treatment phase 3 weeks, cultivation phase 5 weeks
2	Effect of different Na^+^ and K^+^ salts	NaCl, NaNO_3_, NaNO_2_, Na_2_CO_3_ (equivalent to 4.0 g L^−1^ Na^+^); KCl, KNO_3_, KNO_2_, K_2_CO_3_ (equivalent to 6.8 g L^−1^ K^+^)	Treatment phase 2 weeks, cultivation phase 5 weeks
3	Comparison of heavy metal tolerance and accumulation	MnSO_4_ (equivalent to 200, 500, 1000 mg L^−1^ Mn), CdSO_4_ (equivalent to 5, 20, 100 mg L^−1^ Cd), ZnSO_4_ (equivalent to 200, 500, 1000 mg L^−1^ Zn), Pb(NO_3_)_2_ (equivalent to 100, 200, 500 mg L^−1^ Pb)	Treatment phase 2 weeks, cultivation phase 5 weeks
4	Comparison of Pb nitrate and acetate	Pb(NO_3_)_2_, Pb(CH_3_COO)_2_ (equivalent to 200, 500, 1000 mg L^−1^ Pb)	Treatment phase 2 weeks, cultivation phase 7 weeks

**Table 2:** Bioconcentration factors for Na^+^ in different parts of *Ranunculus sceleratus* plants dependent on substrate Na^+^ concentration.

Na^+^ (g L^−1^)	Roots	Rosette Leaf Petioles	Rosette Leaf Blades	Stem	Stem Leaves	Flowers
0.05	174	107	93	91	43	43
0.1	121	68	59	56	27	22
0.2	96	43	35	39	15	18
0.5	25	24	15	18	7	7
1	13	23	11	14	8	6
2	8	14	6	9	6	5
5	5	8	6	7	7	3
10	5	7	2	9	5	3

**Table 3:** Bioconcentration factors for heavy metals in different parts of *Ranunculus sceleratus* plants dependent on metal concentration in substrate.

Metal	Concentration (mg L^−1^)	Roots	Leaves	Stem	Flowers
Mn	200	3.3	18.3	6.0	3.6
	500	2.5	14.1	5.4	3.6
	1000	1.5	7.5	3.2	2.0
Cd	5	20.0	1.8	0.10	0.33
	20	6.9	1.0	0.08	0.38
	100	6.8	0.8	0.05	0.24
Zn	200	19.7	6.7	2.8	1.76
	500	11.7	11.0	1.9	0.96
	1000	8.2	7.0	2.3	0.44
Pb	100	6.0	2.3	0.16	0.03
	200	3.9	1.7	0.12	0.01
	500	2.9	1.4	0.09	0.02

**Table 4:** Bioconcentration factors for Pb in different parts of *Ranunculus sceleratus* plants dependent on salt type and on metal concentration in substrate.

Salt	Concentration (mg L^−1^)	Roots	Small Leaves	Rosette Leaves	Stem	Stem Leaves	Flowers
Pb nitrate	200	4.2	0.87	0.83	0.12	0.09	0.06
	500	4.0	0.77	0.48	0.08	0.03	0.02
	1000	3.7	0.66	0.40	0.05	0.08	0.02
Pb acetate	200	4.1	1.25	1.83	0.12	0.05	0.09
	500	3.4	1.11	0.52	0.08	0.07	0.06
	1000	3.1	2.37	0.95	0.06	0.17	0.01

## Data Availability

All data reported here are available from the authors upon request.

## Supplementary Materials

The following supporting information can be downloaded at: https://www.mdpi.com/article/10.3390/life12121959/s1, Figure S1: *Ranunculus sceleratus* plants in natural habitat; Figure S2: Typical *Ranunculus sceleratus* plants in Experiment 1, 5 weeks after full treatment with NaCl; Figure S3: Crystalline NaCl deposition on leaf surface of *Ranunculus sceleratus* plants treated with 10 g L^−1^ Na^+^ in Experiment 1, 5 weeks after full treatment with NaCl; Figure S4: Typical *Ranunculus sceleratus* plants in Experiment 2, 2 (above) and 4 (below) weeks after full treatment; Figure S5: Typical *Ranunculus sceleratus* plants in Experiment 3, 5 weeks after full treatment; Figure S6: Typical *Ranunculus sceleratus* plants in Experiment 4. From top to bottom: 1, 3, 4, 7 weeks after full treatment.
