# Supplementary material for: Salinity and Heavy Metal Tolerance, and Phytoextraction Potential of Ranunculus sceleratus Plants from a Sandy Coastal Beach
Source: Life (Basel). 2022 Nov 23;12(12):1959. doi: 10.3390/life12121959 (PMC9782882; doi:10.3390/life12121959)
Supplement: Supplementary file 1 [file life-12-01959-s001.zip › life-2034454-supplementary.pdf]

Salinity and heavy metal tolerance, and phytoextraction potential of *Ranunculus sceleratus* plants from sandy coastal beach

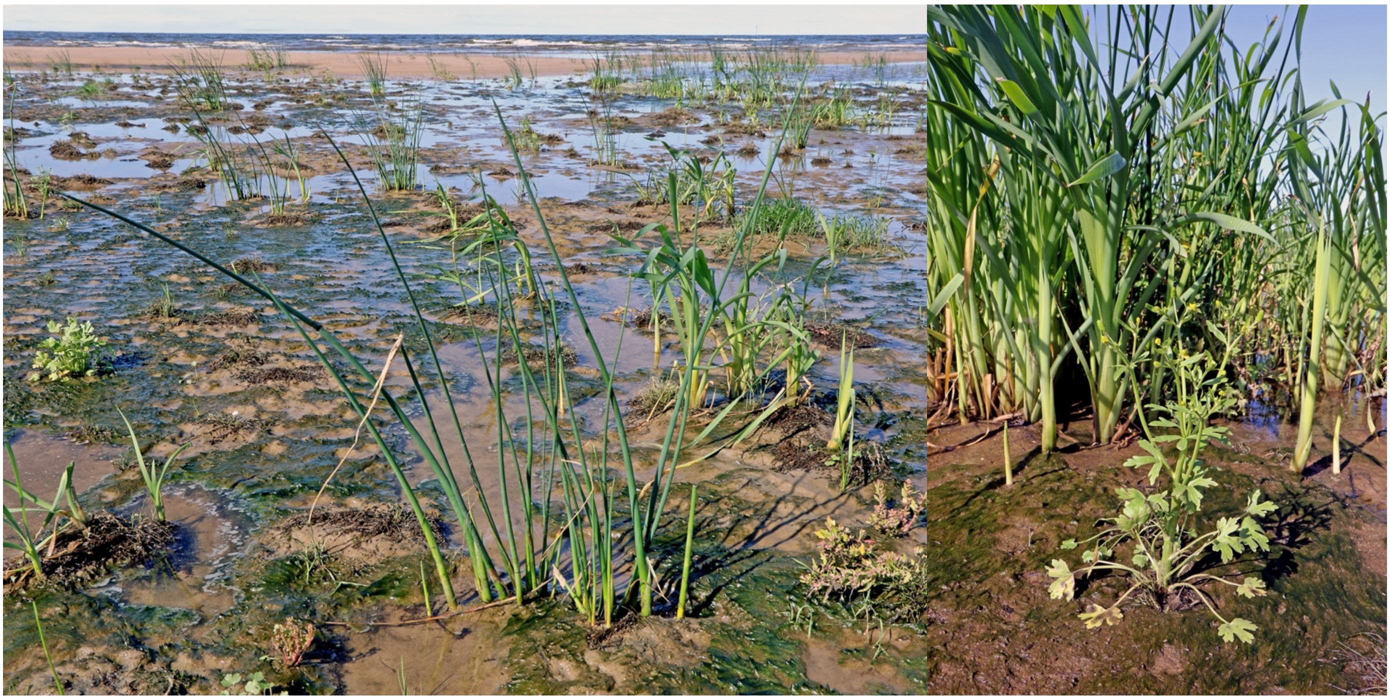

Figure S1. *Ranunculus sceleratus* plants in natural habitat.

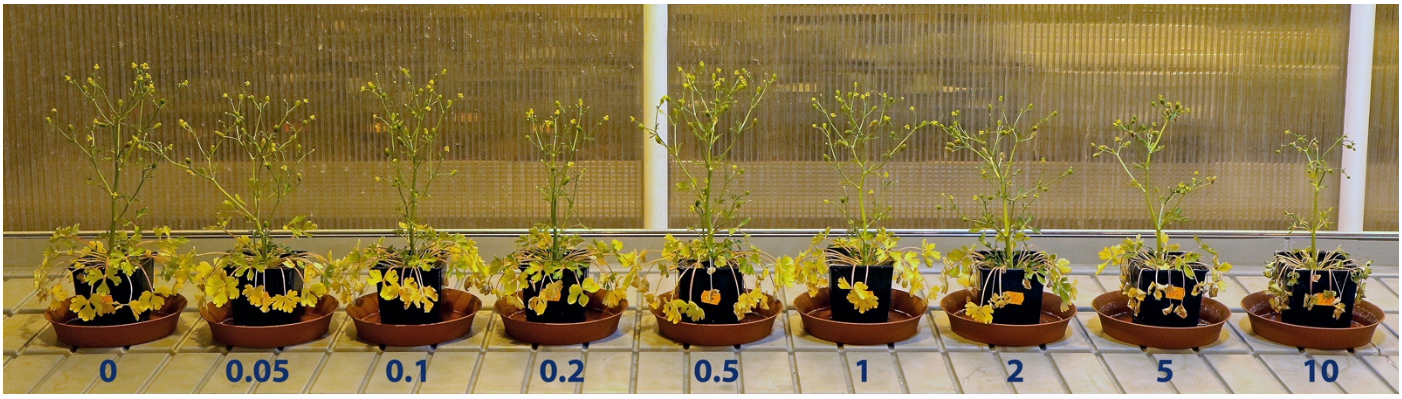

Figure S2. Typical *Ranunculus sceleratus* plants in Experiment 1, 5 weeks after full treatment with NaCl. Numbers indicate amount of Na^+ (g L^{-1}) added in a form of NaCl.

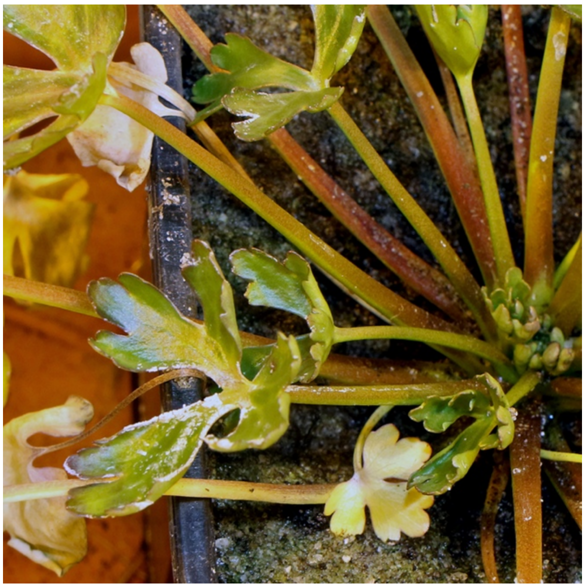

Figure S3. Crystalline NaCl deposition on leaf surface of *Ranunculus sceleratus* plants treated with 10 g L^{-1} Na^+ in Experiment 1, 5 weeks after full treatment with NaCl.

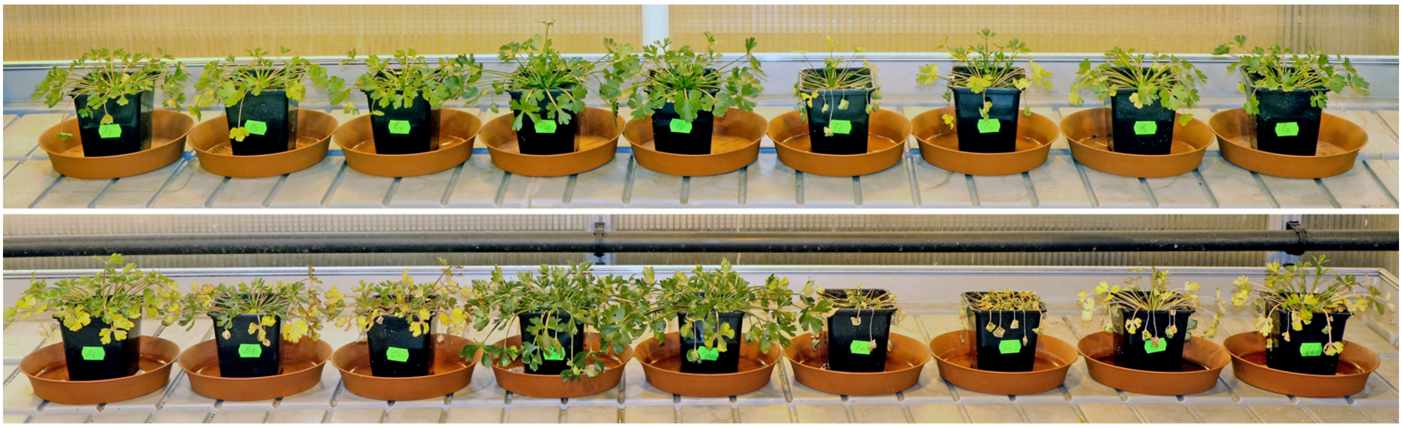

Figure S4. Typical *Ranunculus sceleratus* plants in Experiment 2, 2 (above) and 4 (below) weeks after full treatment. From left to right: control, NaCl, KCl, $NaNO_3$, KNO_3, $NaNO_2$, KNO_2, Na_2CO_3, K_2CO_3. All treatments contained 4.0 g L^{-1} Na^+ or 6.8 g L^{-1} K^+.

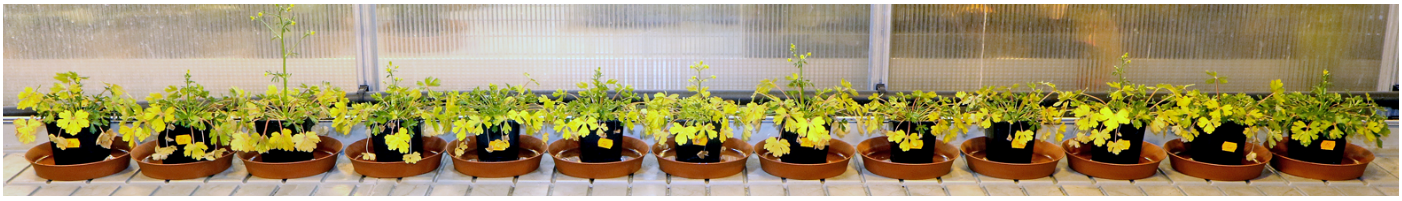

Figure S5. Typical *Ranunculus sceleratus* plants in Experiment 3, 5 weeks after full treatment. From left to right: control, Mn 200 mg L^{-1}, Mn 500 mg L^{-1}, Mn 1000 mg L^{-1}, Cd 5 mg L^{-1}, Cd 20 mg L^{-1}, Cd 100 mg L^{-1}, Zn 200 mg L^{-1}, Zn 500 mg L^{-1}, Zn 1000 mg L^{-1}, Pb 100 mg L^{-1}, Pb 200 mg L^{-1}, Pb 500 mg L^{-1}. Salts used for treatments were $MnSO_4$, $CdSO_4$, $ZnSO_4$ and $Pb(NO_3)_2$).

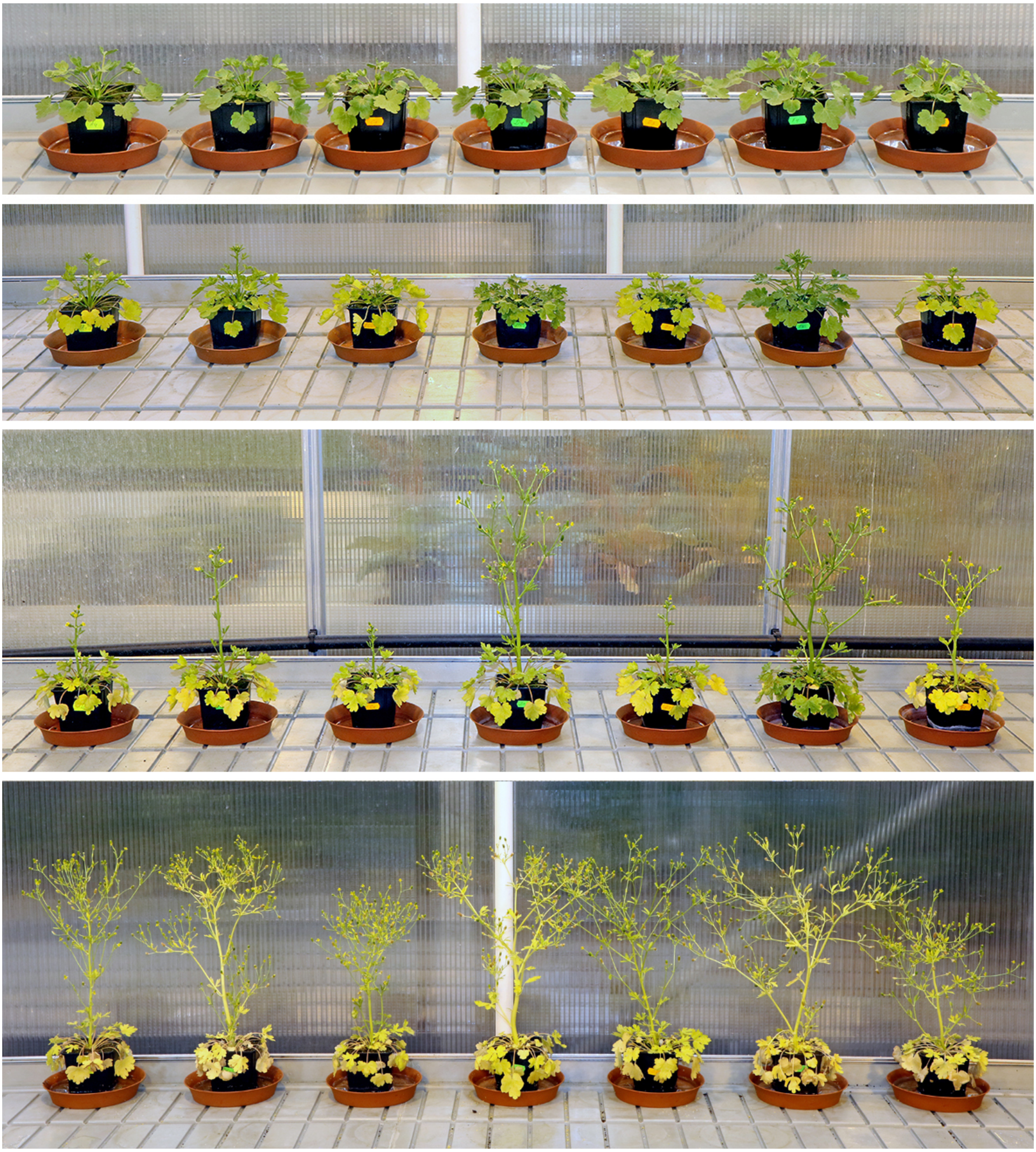

Figure S6. Typical *Ranunculus sceleratus* plants in Experiment 4. From top to bottom: 1, 3, 4, 7, weeks after full treatment. From left to right: control, 200 mg L^{-1} Pb as nitrate, 200 mg L^{-1} Pb as acetate, 500 mg L^{-1} Pb as nitrate, 500 mg L^{-1} Pb as acetate, 1000 mg L^{-1} Pb as nitrate, 1000 mg L^{-1} Pb as acetate.
